# Dietary and Plasma Phospholipid Profiles in Vegans and Omnivores—Results from the RBVD Study

**DOI:** 10.3390/nu14142900

**Published:** 2022-07-14

**Authors:** Juliane Menzel, Alessa Longree, Klaus Abraham, Matthias B. Schulze, Cornelia Weikert

**Affiliations:** 1 Department of Food Safety, German Federal Institute for Risk Assessment, 10589 Berlin, Germany; alessa.longree@gmail.com (A.L.); klaus.abraham@bfr.bund.de (K.A.); cornelia.weikert@bfr.bund.de (C.W.); 2 Institute of Social Medicine, Epidemiology and Health Economics, Charité-Universitätsmedizin Berlin, Corporate Member of Freie Universität Berlin and Humboldt-Universität zu Berlin, 10117 Berlin, Germany; 3 Department of Molecular Epidemiology, German Institute of Human Nutrition Potsdam–Rehbruecke, 14558 Nuthetal, Germany; mschulze@dife.de; 4 Institute of Nutritional Science, University of Potsdam, 14558 Nuthetal, Germany

**Keywords:** SFA, TFA, MUFA, PUFA, n-3 fatty acid, n-6 fatty acid, fatty acids, vegan diet

## Abstract

Over the last few years, the vegan diet has become increasingly popular in Germany. It has been proposed that this diet is generally lower in fat, but less is known about the impact on fatty acid (FA) profiles. Therefore, the cross-sectional “Risks and Benefits of a Vegan Diet” (RBVD) study (*n* = 72) was used to investigate dietary FA intake as well as plasma phospholipid FA in vegans (*n* = 36) compared to omnivores (*n* = 36). Vegans had a significantly lower dietary intake of total fat (median 86 g/day, IQR 64–111) in comparison to omnivores (median 104 g/day, IQR 88–143, *p* = 0.004). Further, vegans had a lower intake of saturated fatty acids (SFA) (*p* < 0.0001) and monounsaturated fatty acids (MUFA) (*p* = 0.001) compared to omnivores. Vegans had a higher intake in total polyunsaturated fatty acids (PUFA), omega-3 and omega-6 PUFA compared to omnivores, but without statistical significance after Bonferroni correction. According to plasma phospholipid profiles, relatively lower proportions of SFA (*p* < 0.0001), total trans fatty acids (TFA) (*p* = 0.0004) and omega-3-FA (*p* < 0.0001), but higher proportions of omega-6-FA (*p* < 0.0001) were observed in vegans. With the exception of omega-3 PUFA, a vegan diet is associated with a more favorable dietary fat intake and more favorable plasma FA profiles and therefore may reduce cardiovascular risk.

## 1. Introduction

In recent years, plant-based diets, i.e., vegetarian or vegan diets, have become increasingly popular as a means to reduce the environmental footprint of food consumption and promote human health and animal welfare [1]. Though the proportions of vegetarians and vegans have been relatively low compared to omnivores [1], their numbers have increased significantly in the last years in Germany and many other Western countries [2]. In particular, an expanding dietary shift toward a vegan diet can be noted, referring to a diet characterized by the abstention of animal products, i.e., meat, fish, dairy and eggs. A vegan diet is based on the consumption of grains, legumes, vegetables, fruits, nuts and seeds. To date, it has been proposed that a vegan diet is generally lower in fat. However, less is known about the impact on fatty acid profiles. Fatty acids (FA) play several important roles in metabolic homeostasis, serving as precursors of signaling molecules, energy sources, and components of membranes and functional lipids [3]. In addition, scientific evidence has demonstrated the important influence of dietary and circulating FA in health and disease; FA are implicated in chronic diseases such as cardiovascular disease, cancer and autoimmune diseases [4]. Interestingly, scientific evidence suggests that a vegan or vegetarian diet may protect against many chronic diseases, e.g., cardiovascular diseases [5]. Linking this, it is expected that the low saturated and high unsaturated fat contents of a plant-based diet may lower CVD risk by improving the blood lipid profile [6]. Indeed, a meta-analysis provided evidence that a shift toward polyunsaturated fatty acids (PUFA) consumption in place of saturated fatty acids (SFA) reduces the risk of coronary heart diseases in randomized controlled trials [7], with similar results observed in prospective cohort studies [7]. Further, prospective cohort studies have supported the role of PUFA, especially omega-3 polyunsaturated FA, e.g., eicosapentaenoic acid (EPA) and docosahexaenoic acid (DHA), for primary prevention of atherosclerotic cardiovascular diseases, although randomized controlled trials often reported no effect [8]. Because fatty fish and fish oil are the most concentrated sources of EPA and DHA, vegans might have a low or inadequate intake of omega-3 PUFA [9]. Interestingly it was shown that non-fish eaters, e.g., vegetarians or vegans, have much lower dietary intakes of EPA and DHA than fish eaters, while the plasma omega-3 PUFA status is higher than expected [9]. To date, only a few studies have investigated the dietary and plasma FA profiles of vegans. Therefore, the present study aimed to investigate dietary FA intake and plasma phospholipid FA profiles in vegans compared to omnivores.

## 2. Materials and Methods

### 2.1. Study Population

Study participants of the “Risks and Benefits of a Vegan Diet” (RBVD) study were recruited by advertisement and examined in Berlin from January to July 2017 [10]. Individuals interested in this announcement contacted the study center at the German Federal Institute for Risk Assessment (BfR) via phone or email. In the following phone call, the study design was briefly explained, and inclusion and exclusion criteria were checked [10]. Inclusion criteria were age 30–60 years and following their current diet for at least one year [10]. Participants with BMI ≥ 30 kg/m^2^, cardiovascular disease, type 2 diabetes, cancer and current infection as well as those who were pregnant or breastfeeding were excluded [10]. An omnivorous diet was defined as the consumption of at least three servings of meat per week or two portions of meat and two servings of processed meat per week [10]. A vegan diet was defined as no consumption of any animal food products [10]. Each participant visited the study center two times [10]. On their first visit, participants gave their written informed consent and received instructions to document their diet using a three-day weighed food record [10]. At the second visit, anthropometric measurements and lifestyle characteristics were assessed, and a fasting blood sample was collected [10]. In total, the final study population comprised 36 vegans and 36 omnivores, who were matched by sex and age [10]. The study was approved by the Ethics Committee of the Charité-Universitätsmedizin Berlin (No. EA4/121/16) and conducted in accordance with the Declaration of Helsinki.

### 2.2. Assessment of Lifestyle Characteristics

Trained personnel collected anthropometric measurements (weight, height, and waist circumference), with participants wearing only light underwear and no shoes. Body weight was assessed by an electronic digital scale (Omron BF511, Omron Healthcare Ltd., Kyoto, Japan), and height was measured using a flexible anthropometer (SECA, Hamburg, Germany). Waist circumference was defined as the horizontal plane midway between the lowest ribs and the iliac crest. Information on physical activity, educational level, and smoking status was assessed by computer-based questionnaires. The educational level was defined as ‘low education’ (no degree), ‘intermediate education’ (vocational school, technical college) or ‘high education’ (university, university of applied sciences). The time spent on cycling, sports, and gardening was determined for summer and winter separately (hours/week). Physical activity comprised the sum of average hours in summer and winter per week of these activities. Walking comprised the sum of average hours per week during summer and winter. Dietary habits, including alcohol consumption and fat intake, were assessed by three-day weighed food records for two weekdays and one weekend day. These data were merged with the German national food code (Bundeslebensmittelschlüssel Version 3.02, BLS) to assign with macro- and micronutrients.

### 2.3. Blood Collection and Laboratory Analysis

About 60 mL of venous blood was collected from fasting participants at the BfR study center. About half of the blood was fractionated and aliquoted into serum, EDTA plasma and erythrocytes and stored in freezers (−80 °C) until analysis. In October 2017, the German Institute of Human Nutrition in Potsdam-Rehbrücke analyzed FA in plasma phospholipids using methods described elsewhere [11]. Briefly, we measured FA using total lipid extraction with *tert*-butyl methyl ether/methanol, solid-phase separation, hydrolysis and methylation with trimethyl sulfonium hydroxide and subsequently analyzed samples by gas chromatography with a flame ionization detector. The FA were identified by reference to standard FA mixtures (37-component FAME mixture, SupelcoTM) run on the same column under identical conditions. All peaks of FA were calculated, and FA composition was expressed as an area percentage of each FA relative to the total area of all detected FA.

Phenotypic markers of stearoyl-CoA-desaturase (SCD-1) activity were used as surrogate measures of tissue SCD-1 activity based on the ratios of relevant product and precursor FA, as follows: C16:1n7c/16:0 (SCD-16) and C18:1n9/18:0 (SCD-18). Further, C18:3n−6/18:2n−6 reflect Δ6-desaturase activity, and C20:4n−6/20:3n−6 reflect Δ5-desaturase activity.

### 2.4. Statistical Analysis

Normally distributed variables were reported as mean and standard deviation (SD). Skewed variables were reported as median and interquartile range (IQR). Categorical variables were reported as a percentage. To compare vegans and omnivores, a Student’s *t*-test (normally distributed variables) or Mann–Whitney U test (not normally distributed variables) was used, and a Chi square test was used for categorical variables.

Analysis of variance (ANOVA) was used to investigate the associations between plasma phospholipid FA and a vegan diet in comparison to an omnivorous diet without adjustment (Model 1). Moreover, multivariable-adjusted analysis of covariance (ANCOVA) was used to further assess the relationship between plasma phospholipid FA and diet status, adjusted for alcohol intake, educational status, physical activity and smoking status (Model 2). Since most of the measured FA were skewed, the variables were log-transformed for ANOVA or ANCOVA. Afterwards, they were back-transformed and expressed as geometric means and 95% confidence intervals (95%-CI). Moreover, sensitivity analyses were performed, analyzing FA profiles after excluding participants who consumed omega-3 FA supplements regularly.

The statistical analyses were performed using SAS software, version 9.4 (SAS institute, Cary, NC, USA). Test findings with *p*-values of <0.05 were considered statistically significant. However, for multiple comparisons, Bonferroni correction was applied for dietary FA (*p* ≤ 0.0013) and for plasma phospholipid FA (*p* ≤ 0.0017). The results reported in this article are based on the dissertation of Dr. Alessa Longree [12].

## 3. Results

Table 1 shows the basic characteristics of the study population in terms of a vegan (*n* = 36) or an omnivorous diet (*n* = 36). The median duration of veganism was 4.8 years (IQR: 3.1–8.7). Due to the sex- and age-matched inclusion of the participants, we observed no differences in sex and age (Table 1). Moreover, no statistically significant differences in anthropometric measurements, physical activity, smoking, education or alcohol consumption were observed between the groups (all *p* > 0.05). However, omnivorous men tended to have a higher alcohol consumption.

### 3.1. Dietary Fatty Acid Intake

Our study analyzed and compared the daily intake of total fat and individual FA of the participants based on the information from three-day weighed food records. The total energy intake in calories did not differ significantly between vegans (median 2270 kcal, IQR 1800–2762) and omnivores (median 2386 kcal, IQR 2081–2737, *p* = 0.3). However, the total fat intake was higher in omnivores (median 104 g/day, IQR 88–143) compared to vegans (median 86 g/day, IQR 64–111, *p* = 0.004). Indeed, as depicted in Table 2, vegans had a lower percentage of total energy intake (%TEI) according to total dietary fat (*p* = 0.0002), as well as SFA (*p* < 0.0001) and monounsaturated fatty acids (MUFA) (*p* = 0.005) in comparison to omnivores. Concerning PUFA, vegans had higher %TEI compared to omnivores (*p* < 0.0001).

As shown in Table 3, vegans had significantly lower dietary intakes of total SFA compared to omnivores (*p* < 0.0001) and lower intakes of individual SFA C4:0-C18:0 (all *p* < 0.0001). The highest intake was observed for palmitic acid in comparison to the other SFA in both dietary groups. Regarding arachidic acid (C20:0), we observed a higher intake in omnivores (median: 305 mg/d, IQR: 234–411) compared to vegans (median: 240 mg/d, IQR: 167–332); however, this did not reach statistical significance (Table 3). Moreover, the median intake of behenic acid (C22:0) was higher in vegans but did not reach significance after Bonferroni correction.

Regarding total MUFA, vegans consumed lower amounts compared to omnivores (*p* = 0.001, Table 2). Oleic acid represents the topmost dietary MUFA in both groups; however, omnivores had a higher intake (Table 3) but without statistical significance after Bonferroni correction.

The present study revealed that vegans had a higher intake of total PUFA (*p* = 0.002) and sum of omega-3 FA (*p* = 0.03) compared to omnivores; however, this did not reach significance after Bonferroni correction (Table 3). With respect to single omega-3 FA, α-linolenic acid (C18:3n3, ALA, the mainly consumed FA) was associated with a higher intake in vegans compared to omnivores (*p* = 0.0002). Moreover, we detected a higher intake of EPA (median: 41 mg/d, IQR: 26–186) and DHA (median: 126 mg/d, IQR: 45–275) in omnivores compared to vegans (EPA median: 0.8 mg/d, IQR: 0.1–2.9; DHA median: 7.3 mg/d, IQR: 2.9–23, both *p* < 0.0001). As depicted in Table 3, similar results regarding omega-6 FA were observed. Accordingly, vegans consumed higher amounts of total omega-6 FA (*p* = 0.005). Linoleic acid (C18:2n6c, LA) was consumed as the topmost omega-6 FA, with a significantly higher amount in vegans (median: 17,170 mg/d, IQR: 13,108–25,354) compared to omnivores (median: 11,382 mg/d, IQR: 8632–16,804, *p* = 0.003). Both differences were no longer significant after correction for Bonferroni. Further, our study noticed that omnivores (median: 166 mg/d, IQR: 115–240) had a higher intake of arachidonic acid (AA) compared to vegans (median: 5.5 mg/d, IQR: 3.6–11, *p* < 0.0001). Dietary omega-6 to omega-3 ratio did not differ significantly between vegans (5.9:1, IQR: 4.5:1–7.5:1) and omnivores (5.3:1, IQR: 4.5:1–7.3:1, *p* = 0.8, Table S1).

### 3.2. Fatty Acids in Plasma Phospholipids

As demonstrated in Table 4, vegans had significantly lower proportions of total SFA and of individual SFA pentadecanoic acid (C15:0), palmitic acid (C16:0) and heptadecanoic acid (C17:0) (*p* < 0.0001). However, arachidic acid (C20:0) was significantly higher in vegans (*p* < 0.0001). After adjusting for the main lifestyle factors in Model 2, results for C15:0, C16:0, C17:0, C20:0 and total SFA remained significant.

Regarding total plasma phospholipid proportions of total MUFA, our study observed no difference between vegans and omnivores. Within this FA group, the oleic acid (C18:1n9c) is predominant in both dietary groups (Table 4).

Proportions of total omega-3 FA were significantly lower in vegans (*p* < 0.0001). We could not detect any difference in the proportion of the essential omega-3 FA ALA between vegans and omnivores (Table 4, *p* = 0.26 in Model 2). However, the amount of EPA and DHA was significantly higher in omnivores compared to vegans (both *p* < 0.0001).

Total omega-6 FA and the essential omega-6 FA LA reached significantly higher proportions in vegans in both models (both *p* < 0.0001). There were also higher proportions of eicosadienoic acid (C20:2n6) in vegans (*p* = 0.0009 in Model 2). We could not demonstrate any difference between the dietary groups in both models with regards to the proportion of γ-linoleic acid (C18:3n6), dihomo-γ-linolenic acid (C20:3n6), arachidonic acid (C20:4n6) and adrenic acid (C22:4n6) (Table 4). Regarding total PUFA in plasma phospholipids, we observed no difference between vegans and omnivores (*p* = 0.11 in Model 2). Plasma omega-6 to omega-3 ratio was significantly different between vegans (median 12.1:1, IQR: 10.5:1–14.6:1) in comparison to omnivores (6.9:1, IQR: 6.0:1–8.3:1, *p* < 0.0001, Table S1).

Regarding total TFA, vegans had significantly lower proportions (*p* = 0.0004 in Model 2), but no difference could be found for the principle TFA elaidic acid (C18:1n9t) (*p* = 0.82 in Model 2). Regarding sensitivity analyses, we repeated the comparison of plasma phospholipid FA proportions in vegans and omnivores after excluding participants who consume omega-3 FA supplements (*n* = 3) regularly. Accordingly, we detected no relevant differences in the results (data not shown).

Table 5 depicts the results of the estimated desaturase computations. A significant difference in enzyme activity can only be seen for SCD-1 C16:0, which was higher in omnivores, but lost significance after adjusting for lifestyle factors (Model 2). No differences were observed for Δ5-desaturase, Δ6-desaturase or SCD-1 C18:0.

## 4. Discussion

In this study, we assessed dietary FA intake as well as plasma phospholipid FA profiles in vegans and omnivores. We detected lower total fat, SFA and MUFA intake in vegans. Vegans had a higher intake in total PUFA, omega-3 and omega-6 PUFA compared to omnivores, but without statistical significance after Bonferroni correction. In plasma phospholipids, more favorable proportions of FA were seen in vegans with lower SFA and TFA and higher total omega-6-FA compared to an omnivorous diet. On the other hand, vegans had significantly lower proportions regarding long-chain omega-3 FA, but no difference was seen for ALA.

### 4.1. Saturated Fatty Acids

SFA mainly occur in animal products, but they are also found in a few plant foods, mostly in tropical fats and oils, such as coconut oil, palm oil and palm kernel oil [13]. The German Nutrition Society recommends, depending on the level of physical activity, a fat intake limited to 30 to 35% of daily energy intake; SFA should account for less than 10% of daily energy intake [14]. Our results showed that omnivores (total fat mean: 42.8%TEI) clearly exceeded the dietary recommendations of daily total fat intake (vegans total fat %TEI mean: 35.4). In addition, with regards to SFA, omnivores (SFA mean: 17.7%TEI) exceeded the recommendations, but vegans met the recommendations (vegans SFA mean: 7.0%TEI).

In line with other studies [2,15,16,17,18,19,20], our study revealed lower dietary intakes of total SFA in vegans compared to omnivores. This is not surprising since a western diet is rich in meat and other animal products, which are a rich source of SFA [21].

Regarding plasma FA, vegans also had significantly lower plasma phospholipid proportions of SFA, which corresponds with previous findings [17,18]. Interestingly, regarding individual plasma SFA, C15:0 and C17:0 were also found in plasma phospholipids in vegans taking part in the present study, albeit lower than in omnivores. In line with this, other studies also detected concentrations of odds-chain FA in vegans. Allen et al. found odd-chain FA in plasma phospholipids in humans on a vegan diet, albeit less than in omnivores and vegetarians [22]. Further, concentrations of C17:0 in red blood cells of vegans were found to be as high as in dairy fat consumers [23]. In the past, C15:0 and C17:0 might have been wrongly attributed to dairy product intake alone [24]. Rather, they can be synthesized endogenously, either by elongation of shorter odd-chain SFA or by chain shortening of very-long-chain FA [24,25]. C15:0 might also be converted from phytosphingosine, the sphingoid base of glycosphingolipids [25]. Accordingly, recent study findings from the EPIC-Potsdam study also noticed a heterogeneous integration of odd-chain FA in different lipid classes, i.e., triacylglycerols, free FA, cholesteryl esters, and phosphatidylcholines [26]. This suggests that odd-chain FA are subject to complex metabolic regulation [26].

### 4.2. Monounsaturated Fatty Acids

MUFA are mostly found in olives, avocados, nuts such as almonds, hazelnuts, and pecans, and their respective oils, as well as in canola and safflower oils, and are found in high concentrations in seeds such as pumpkin and sesame seeds [13]. Nevertheless, MUFA can also come from animal-based sources like red and processed meats, as well as high-fat dairy products [27,28]. The German Nutrition Society recommends a daily intake of 10–15% of energy from MUFA [14]. We demonstrate that vegans meet the recommendations (MUFA mean: 12.3%TEI) and omnivores almost meet the recommendations (MUFA mean: 15.3%TEI). MUFA were consumed in a significantly lower amount in vegans compared to omnivores. Previous studies on dietary MUFA intake show mixed results. On the one hand, scientific evidence showed that vegans have lower dietary intakes of MUFA compared to omnivores [2,15,18,19], while other studies could not detect any significant difference in dietary MUFA intake between vegans and omnivores [16,17]. The source of dietary intake of MUFA and possible dietary regional differences might provide an explanation for the mixed results. In fact, if the main consumption of MUFA comes from plant-based sources, no difference would thus be expected between vegans and omnivores. However, if the dietary intake of MUFA mainly comes from animal-based sources in a population, a difference between vegans and omnivores might be expected to a larger extent. It has been proposed that oleic acid represents the topmost MUFA provided in the diet [29]; this was also seen in the present study.

We did not discover any difference regarding total plasma phospholipid proportions of MUFA, but we found significant differences for individual plasma MUFA proportions. Indeed, Roselle et al. showed that vegans had significantly lower levels of palmitoleic acid (C16:1n7) (*p* < 0.001), whereas levels of oleic acid (C18:1n9) did not differ significantly [18]. This in line with our study, which shows significantly lower proportions of palmitoleic acid in vegans in comparison to omnivores, whereas oleic acid did not differ between the two groups.

### 4.3. Polyunsaturated Fatty Acids

PUFA can be found in different oils, such as safflower, sunflower or corn oils, but also in soybeans, walnuts, flax seeds and fish [13]. On average, vegans received 10.1% of their daily energy from PUFA in our study, whereas omnivores received only 6.2%TEI. The German Nutrition Society recommends up to 10% of PUFA from total energy [14].

In line with our study, other studies [2,15,16,18,19] also showed a higher intake of total PUFA in vegans compared to omnivores. For example, in a Finnish study, vegans had higher PUFA intake compared to omnivores (26% ± 11% vs. 7.8% ± 2.4% from energy) [17]. In our study, vegans had higher plasma proportions of total PUFA, although the difference was not significant after Bonferroni correction.

#### 4.3.1. Omega-3 Polyunsaturated Fatty Acids

The present study demonstrates a higher intake of total omega-3 FA (although not statistically significant after Bonferroni correction) in vegans compared to omnivores, which is largely due to the high dietary intake of ALA, in addition to the low intake of DHA and EPA. Indeed, ALA is the major plant-based omega-3 PUFA and is found in walnuts, flaxseeds, hemp seeds and their oils as well as in rapeseed (canola) oil and in smaller amounts in soya oil and green, leafy vegetables [9]. In line with our findings, a review by Burns-Whitmore et al. noticed that the majority of the included studies showed a high intake of ALA in vegans compared to meat eaters/omnivores [30]. Only a few studies observed lower intakes of ALA in vegans or non-significant differences of ALA compared to meat eaters/omnivores [30]. On the other hand, this review revealed that most studies showed significantly low to missing dietary intakes of DHA and EPA in vegans, unless vegans took algal supplements [30]. With the exception of algae, DHA and EPA are mostly found in seafood, including fatty fish and fish oil. Therefore, vegans might rely on endogenous synthesis to obtain the long chain omega-3 PUFA DHA and EPA. ALA is endogenously converted to DHA and EPA by the elongation and desaturation enzymes, but it is reported that ALA is very sparingly and inefficiently converted into long-chain omega-3 FA DHA and EPA [31]. Further, it is well known that an excess of LA competitively interferes with the ability of ALA to utilize the elongation and desaturase enzymes, thereby suppressing the conversion of ALA to EPA and EPA to DHA [30]. It is suspected that the human conversion rate of ALA to EPA and DHA is about 5–8% [30]. However, a preferential affinity to omega-3 PUFA has been reported [8]. Moreover, recent research indicates that this could be more variable [9]. Indeed, it has been suggested that greater conversion could occur in individuals consuming less EPA and DHA [9]. Accordingly, our study shows that vegans had a very low intake of EPA and DHA but a respectable plasma proportion of EPA and DHA. In addition, our study detected no difference in concentrations of ALA between vegans and omnivores, while the dietary intake of ALA was higher in vegans. Therefore, we might suggest that the endogenous synthesis contributes substantially to the conversation to EPA and DHA following a vegan diet. However, in most studies, both serum and plasma levels of EPA and DHA are lower in vegans compared to non-vegans [30]. Regarding ALA, some studies noticed higher ALA plasma in vegans compared to meat eaters [24,25], while the majority of the studies observed no differences in ALA plasma/serum levels between vegans and non-vegans [9,17,18]. Accordingly, Burns-Whitmore et al. [30] suggested that this variability in plasma/serum ALA may reflect the short-term ALA status, while the levels of EPA and DHA in plasma and serum may reflect long-term omega-3 status in vegans. In addition, it has been proposed that ALA from the diet can be made available by incorporation into cell membranes and pools for storage (mainly in adipose tissue), energy production, or conversion to EPA and DHA [32], and therefore they may no longer be traced in plasma phospholipids.

#### 4.3.2. Omega-6 Polyunsaturated Fatty Acids

Omega-6 FA are mostly found in corn and soybean oils, as well as sunflower, safflower, and cottonseed oils [33]. We noticed a higher dietary intake FA (although not statistically significant after Bonferroni correction) and a significantly higher proportion of plasma phospholipid omega-6 FA in vegans. Looking at individual dietary omega-6 FA, LA was the topmost dietary omega-6 FA in both groups; however, vegans had a higher intake. This is in line with current scientific evidence. Indeed, Burns-Whitmore et al. detected that the majority of the studies were higher in LA dietary intake in vegans compared to non-vegans [30], and no study reported lower LA intakes [30].

Regarding plasma phospholipid FA, vegans had significantly higher proportions of LA. Except for eicosadienoic acid (C20:2n6) and docosapentaenoic acid n-6 (C22:5n6), we observed no difference in other omega-6 PUFA between vegans and omnivores. This is in line with a review mentioning that the majority of the studies observed significantly higher LA but no significant differences of AA in vegans’ serum/plasma compared to non-vegans [30]. Since western diets, especially vegan diets, contain higher amounts of LA than ALA, with both competing for the same desaturases, the conversion might shift to higher conversion rates from LA because of competitive inhibition of Δ5- and Δ6-desaturases [9,34]. Nevertheless, the present study observed no differences of Δ5-desaturase and Δ6-desaturase between vegans and omnivores. Further, as mentioned above, we see no hints of suppressing the conversion of ALA to EPA and EPA to DHA in vegans.

### 4.4. Trans Fatty Acids

The major contribution of TFA in our diet comes from products containing hardened fats [35]. Processed foods and oils provide approximately 80% of trans fats in the diet, compared to 20% that occur naturally in food from animal sources [36]. The major dietary sources of trans fats are cakes, cookies, crackers, animal products, margarine, fried potatoes, potato chips and popcorn [36]. Regarding total plasma TFA, we found significantly lower proportions in vegans. No difference occurred regarding the principal TFA, elaidic acid, the main TFA isomer in industrial hydrogenation [36]. In line with this, Kristensen et al. noticed vegans consume lower amounts of TFA [15].

### 4.5. Strengths and Limitations

Several strengths should be mentioned. The RBVD study provided comprehensive high-quality data as a result of the standardized procedures, including the collection of blood, in combination with extensive information from computer-based questionnaires, a dietary assessment using a 3-day weighed food records and anthropometric measurements. Nevertheless, our study had a relatively small study population, which is seen as a limitation. The cross-sectional design does not allow for causal inference. Moreover, the study included middle-aged men and women from a small area (Berlin, Germany); thus, the results may not be generalizable to other populations. Further, the activity for Δ5- and Δ6-desaturase and stearoyl-coA-desaturase (SCD-16, SCD-18) were estimated by calculating the ratio of the FA product to the FA precursor. This is seen as a limitation, but a direct measurement of desaturase activity is only possible via tissue biopsy, which is not justifiable in healthy individuals [37].

## 5. Conclusions

In conclusion, the present study detected lower total fat, SFA and MUFA intake in vegans. Regarding plasma phospholipids, vegans had lower proportions of SFA and TFA and higher proportions of total omega-6-FA compared to an omnivorous diet. On the other hand, vegans had significantly lower proportions regarding total omega-3 FA. Therefore, a vegan diet is associated with a more favorable dietary fat intake and more favorable plasma FA profiles and thus may reduce risk factors for cardiovascular diseases.

## Figures and Tables

**Table 1:** Characteristics of the study population according to vegans or omnivores.

Characteristics	Vegans (*n* = 36)	Omnivores (*n* = 36)	*p*-Value
Duration vegan diet (years)	4.8 (3.1–8.7)		
Men	50.0% (18)	50.0% (18)	
Age (years)	37.5 (32.5–44.0)	38.5 (32.0–46.0)	0.75
**Anthropometry**			
BMI (kg/m^2^)	22.9 ± 3.2	24.0 ± 2.1	0.08
Waist circumference (cm)			
Women	73.1 ± 6.9	77.2 ± 6.2	0.07
Men	84.5 ± 8.9	86.0 ± 6.1	0.56
**Education (%)**			0.60
Low	0.0% (0)	2.8% (1)	
Intermediate	30.6% (11)	30.6% (11)	
High	69.4% (25)	66.7% (24)	
**Lifestyle**			
Physical activity (h/week)	2.8 (0.88–3.8)	2.3 (1.2–4.1)	0.69
Smoking status			0.30
Non-smoker	66.7% (24)	58.3% (21)	
Ex-smoker	22.2% (8)	16.7% (6)	
Smoker	11.1% (4)	25.0% (9)	
Alcohol consumption (g/day)			
Women	0.10 (0.00–4.69)	0.21 (0.02–4.88)	0.22
Men	0.04 (0.00–2.00)	3.85 (0.36–16.2)	0.09

Variables expressed as a percentage (*n*), mean ± SD, or median (IQR). BMI: body mass index.

**Table 2:** Comparison of %TEI of different fat groups according to vegans or omnivores.

Fat Groups	Vegans (*n* = 36)	Omnivores (*n* = 36)	*p*-Value
Total fat	35.4 ± 8.5	42.8 ± 7.7	0.0002
SFA	7.0 ± 2.9	17.7 ± 4.4	<0.0001
MUFA	12.3 ± 4.8	15.3 ± 4.0	0.005
PUFA	10.1 ± 4.3	6.2 ± 2.4	<0.0001

Variables expressed as a mean ± SD. SFA: saturated fatty acids; MUFA: monounsaturated fatty acids; PUFA: polyunsaturated fatty acids.

**Table 3:** Dietary intake of FA in milligrams per day based on three-day weighed food records.

Characteristics		Vegans (*n* = 36)	Omnivores (*n* = 36)	*p*-Value
**SFA**				
C4:0	Butyric acid	10 (2–67)	1449 (970–2009)	<0.0001 ^a^
C6:0	Capronic acid	15 (3–60)	822 (553–1252)	<0.0001 ^a^
C8:0	Caprylic acid	73 (25–185)	674 (490–823)	<0.0001 ^a^
C10:0	Capric acid	76 (44–174)	1223 (894–1654)	<0.0001 ^a^
C12:0	Lauric acid	351 (190–1099)	2010 (1455–2894)	<0.0001 ^a^
C14:0	Myristic acid	388 (233–808)	4936 (3573–6599)	<0.0001 ^a^
C15:0	Pentadecanoic acid	20 (13–32)	481 (348–636)	<0.0001 ^a^
C16:0	Palmitic acid	8743 (6126–10,558)	20,326 (17,336–26,618)	<0.0001 ^a^
C17:0	Heptadecanoic acid	56 (42–73)	386 (278–477)	<0.0001 ^a^
C18:0	Stearic acid	3169 (2400–4221)	9331 (7112–11,437)	<0.0001 ^a^
C20:0	Arachidic acid	240 (167–332)	305 (234–411)	0.05
C22:0	Beheric acid	126 (82–189)	74 (43–129)	0.004
C24:0	Lignoceric acid	45 (28–66)	35 (24–55)	0.17
Sum SFA		16,140 (10,646–22,269)	42,949 (34,439–54,641)	<0.0001 ^a^
**MUFA**				
C14:1	Myristoleic acid	5.6 (1.6–23)	534 (416–716)	<0.0001 ^a^
C15:1	Pentadecenoic acid	1.1 (0.0–6.2)	185 (136–312)	<0.0001 ^a^
C16:1n7c	Palmitoleic acid	518 (346–677)	1888 (1489–2622)	<0.0001 ^a^
C17:1	Heptadecenoic acid	11 (7- 18)	278 (225–433)	<0.0001 ^a^
C18:1n9c	Oleic acid	26,703 (20,020–34,096)	32,540 (27,286–43,532)	0.005
C20:1	Eicosenic acid	454 (269–656)	443 (255–914)	0.64
C22:1	Eruric acid	72 (33–219)	81 (30–365)	0.42
C24:1	Nervonic acid	13 (3–19)	4.1 (1.2–12)	0.08
Sum MUFA		28,394 (21,877–36,385)	35,950 (30,772–48,656)	0.001 ^a^
**Omega-3 PUFA**				
C18:3n3	α-linolenic acid (ALA)	2855 (1938–4970)	1481 (1222–2483)	0.0002 ^a^
C18:4n3	Stearidonic acid	0.0 (0.0–0.0)	1.2 (0.0–6.0)	<0.0001 ^a^
C20:3	Eicosatrienic acid	1.1 (0.0–5.9)	26 (11–47)	<0.0001 ^a^
C20:5n3	Eicosapentaenoic acid (EPA)	0.8 (0.1–2.9)	41 (26–186)	<0.0001 ^a^
C22:6n3	Docosahexaenoic acid (DHA)	7.3 (2.9–23)	126 (45–275)	<0.0001 ^a^
Sum omega-3 PUFA		2868 (1942–5019)	1962 (1494–3410)	0.03
**Omega-6 PUFA**				
C18:2n6c	Linoleic acid (LA)	17,170 (13,108–25,354)	11,382 (8632–16,804)	0.003
C20:2n6	Eicosadienic acid	7.2 (4.6–11)	21 (12–57)	<0.0001 ^a^
C20:4n6	Arachidonic acid (AA)	5.5 (3.6–11)	166 (115–240)	<0.0001 ^a^
C22:2n6	Docosadienic acid	0.0 (0.0–0.0)	0.0 (0.0–0.3)	0.006
C22:5n6	Docosapentaenoic acid	0.7 (0.0–6.1)	50 (19–119)	<0.0001 ^a^
Sum omega-6 PUFA		17,187 (13,120–25,364)	11,608 (8738–17,320)	0.005
C19:3	Nonadecatrienic acid	0.0 (0.0–1.5)	0.0 (0.0–15.0)	0.08
C22:3	Docosatrienic acid	0.0 (0.0–0.0)	0.0 (0.0–0.0)	0.04
C22:4	Docosatetraenic acid	0.0 (0.0–0.0)	0.0 (0.0–1.0)	0.003
Sum PUFA		21,169 (17,939–28,753)	13,557 (10,500–21,402)	0.002

Data expressed as a median and IQR in mg/d. ^a^ statistically significant after Bonferroni correction for multiple comparisons (threshold is *p* = 0.0013).

**Table 4:** Proportions of FA in plasma phospholipid (%) of vegans and omnivores.

Characteristics			Vegans (*n* = 36)	Omnivores (*n* = 36)	*p*-Value
**SFA**					
C14:0	Myristic acid	Model 1	0.26 (0.24–0.28)	0.29 (0.27–0.31)	0.04
		Model 2	0.26 (0.22–0.31)	0.29 (0.24–0.34)	0.11
C15:0	Pentadecanoic acid	Model 1	0.15 (0.14–0.16)	0.25 (0.24–0.27)	<0.0001 ^a^
		Model 2	0.13 (0.12–0.16)	0.24 (0.21–0.27)	<0.0001 ^a^
C16:0	Palmitic acid	Model 1	28.3 (27.8–28.7)	30.5 (30.0–30.9)	<0.0001 ^a^
		Model 2	28.7 (27.7–29.7)	30.7 (29.7–31.8)	<0.0001 ^a^
C17:0	Heptadecanoic acid	Model 1	0.38 (0.36–0.40)	0.44 (0.42–0.47)	<0.0001 ^a^
		Model 2	0.36 (0.31–0.40)	0.42 (0.37–0.48)	<0.0001 ^a^
C18:0	Stearic acid	Model 1	16.2 (15.8–16.7)	15.5 (15.0–15.9)	0.03
		Model 2	16.3 (15.3–17.5)	15.7 (14.7–16.7)	0.05
C20:0	Arachidic acid	Model 1	0.15 (0.14–0.16)	0.10 (0.09–0.10)	<0.0001 ^a^
		Model 2	0.15 (0.13–0.17)	0.10 (0.09–0.11)	<0.0001 ^a^
Sum SFA		Model 1	45.5 (45.1–46.0)	47.1 (46.6–47.5)	<0.0001 ^a^
		Model 2	46.0 (44.9–47.0)	47.5 (46.5–48.5)	<0.0001 ^a^
**MUFA**					
C16:1n7c	Palmitoleic acid	Model 1	0.33 (0.29–0.37)	0.45 (0.40–0.51)	0.0009 ^a^
		Model 2	0.37 (0.28–0.49)	0.47 (0.36–0.61)	0.01
C18:1n7c	cis-Vaccenic acid	Model 1	1.77 (1.69–1.85)	1.40 (1.34–1.47)	<0.0001 ^a^
		Model 2	1.79 (1.60–1.99)	1.41 (1.27–1.56)	<0.0001 ^a^
C18:1n9c	Oleic acid	Model 1	10.7 (10.2–11.3)	10.6 (10.1–11.2)	0.79
		Model 2	11.6 (10.3–12.9)	11.1 (10.0–12.4)	0.30
C20:1n9	Gondoic acid	Model 1	0.28 (0.26–0.30)	0.17 (0.16–0.18)	<0.0001 ^a^
		Model 2	0.26 (0.22–0.31)	0.16 (0.14–0.19)	<0.0001 ^a^
Sum MUFA		Model 1	13.2 (12.6–13.8)	12.7 (12.1–13.3)	0.25
		Model 2	14.1 (12.7–15.6)	13.2 (12.0–14.5)	0.06
**Omega-3 PUFA**					
C18:3n3	α-Linolenic acid (ALA)	Model 1	0.27 (0.24–0.29)	0.25 (0.22–0.27)	0.37
		Model 2	0.31 (0.24–0.39)	0.28 (0.23–0.35)	0.26
C20:5n3	Eicosapentaenoic acid (EPA)	Model 1	0.49 (0.42–0.56)	0.96 (0.83–1.11)	<0.0001 ^a^
		Model 2	0.48 (0.35–0.67)	0.92 (0.67–1.26)	<0.0001 ^a^
C22:5n3	Docosapentaenoic acid	Model 1	0.70 (0.64–0.76)	0.79 (0.73–0.86)	0.03
		Model 2	0.66 (0.54–0.79)	0.74 (0.62–0.88)	0.06
C22:6n3	Docosahexaenoic acid (DHA)	Model 1	1.50 (1.36–1.65)	2.90 (2.63–3.19)	<0.0001 ^a^
		Model 2	1.35 (1.08–1.69)	2.64 (2.14–3.25)	<0.0001 ^a^
Sum omega-3 PUFA		Model 1	3.02 (2.80–3.26)	4.98 (4.61–5.38)	<0.0001 ^a^
		Model 2	2.82 (2.35–3.38)	4.66 (3.94–5.52)	<0.0001 ^a^
**Omega-6 PUFA**					
C18:2n6c	Linoleic acid (LA)	Model 1	25.7 (24.9–26.5)	21.9 (21.3–22.6)	<0.0001 ^a^
		Model 2	25.4 (23.6–27.2)	21.7 (20.4–23.3)	<0.0001 ^a^
C18:3n6	γ-linoleic acid	Model 1	0.08 (0.07–0.09)	0.08 (0.07–0.09)	0.82
		Model 2	0.09 (0.07–0.13)	0.08 (0.06–0.11)	0.27
C20:2n6	Eicosadienoic acid	Model 1	0.37 (0.34–0.39)	0.30 (0.28–0.33)	0.0005 ^a^
		Model 2	0.34 (0.29–0.40)	0.28 (0.24–0.33)	0.0009 ^a^
C20:3n6	Dihomo-γ-linolenic acid	Model 1	2.55 (2.34–2.77)	2.77 (2.55–3.02)	0.16
		Model 2	2.48 (2.03–3.04)	2.71 (2.24–3.27)	0.18
C20:4n6	Arachidonic acid (AA)	Model 1	8.23 (7.68–8.81)	8.78 (8.20–9.40)	0.18
		Model 2	7.67 (6.56–8.97)	8.45 (7.30–9.78)	0.06
C22:4n6	Adrenic acid	Model 1	0.28 (0.26–0.30)	0.28 (0.26–0.30)	0.96
		Model 2	0.28 (0.24–0.32)	0.28 (0.24–0.32)	0.85
C22:5n6	Docosapentaenoic acid	Model 1	0.16 (0.14–0.18)	0.23 (0.20–0.25)	<0.0001 ^a^
		Model 2	0.17 (0.13–0.21)	0.24 (0.19–0.30)	<0.0001 ^a^
Sum omega-6 PUFA		Model 1	37.8 (37.1–38.4)	34.6 (34.0–35.2)	<0.0001 ^a^
		Model 2	36.6 (35.3–38.0)	34.0 (32.9–35.2)	<0.0001 ^a^
Sum PUFA		Model 1	40.8 (40.3–41.5)	39.7 (39.2–40.3)	0.009
		Model 2	39.4 (38.2–40.6)	38.7 (37.7–39.9)	0.11
**Trans FA**					
C18:1n7t	Vaccenic acid	Model 1	0.05 (0.04–0.06)	0.12 (0.10–0.13)	<0.0001 ^a^
		Model 2	0.05 (0.03–0.06)	0.11 (0.08–0.15)	<0.0001 ^a^
C18:1n9t	Elaidic acid	Model 1	0.13 0.12–0.15)	0.14 (0.13–0.16)	0.38
		Model 2	0.15 (0.12–0.20)	0.16 (0.13–0.20)	0.82
C18:2n6t	Linolelaidic acid	Model 1	0.08 (0.07–0.09)	0.07 (0.06–0.07)	0.0003 ^a^
		Model 2	0.08 (0.07–0.10)	0.07 (0.06–0.08)	0.009
Sum TFA		Model 1	0.27 (0.25–0.29)	0.33 (0.31–0.36)	<0.0001 ^a^
		Model 2	0.28 (0.24–0.33)	0.34 (0.29–0.40)	0.0004 ^a^

All values are presented as geometric means (95%-CI). Model 1: unadjusted; Model 2: adjusted for alcohol intake (g/d), educational level, physical activity (h/week) and smoking status. ^a^ statistically significant after Bonferroni correction for multiple comparisons (threshold is *p* = 0.0017).

**Table 5:** Estimated desaturase activities (product-to-precursor ratio) according to diet group.

Characteristics	Vegans (*n* = 36)	Omnivores (*n* = 36)	*p*-Value
**Δ5-desaturase** (C18:3n−6/18:2n−6)			
Model 1	3.23 (2.90–59)	3.17 (2.85–52)	0.80
Model 2	3.09 (2.41–96)	3.12 (2.47–93)	0.90
**Δ6-desaturase** (C18:1n9/18:0)			
Model 1	0.003 (003–004)	0.004 (003–004)	0.26
Model 2	0.004 (003–005)	0.004 (003–005)	0.76
**SCD-16** (C16:1n7c/16:0)			
Model 1	0.01 (01–01)	0.02 (01–02)	0.006
Model 2	0.01 (01–02)	0.02 (01–02)	0.06
**SCD-18** (C18:1n9/18:0)			
Model 1	0.66 (62–70)	0.69 (64–73)	0.43
Model 2	0.71 (61–82)	0.71 (62–28)	0.91

Variables are presented as a geometric mean (95%-CI). Model 1: unadjusted; Model 2: adjusted for alcohol intake (g/day), educational level, physical activity (h/week) and smoking status.

## Data Availability

The datasets generated and/or analyzed during the current Risks and Benefits of a Vegan Diet study are not publicly available due to provisions of the data protection regulations.

## Supplementary Materials

The following supporting information can be downloaded at: https://www.mdpi.com/article/10.3390/nu14142900/s1, Table S1: Omega-6 to omega-3 ratios according to diet group and sex.
