# Supplementary material for: Dietary and Plasma Phospholipid Profiles in Vegans and Omnivores—Results from the RBVD Study
Source: Nutrients. 2022 Jul 14;14(14):2900. doi: 10.3390/nu14142900 (PMC9320578; doi:10.3390/nu14142900)
Supplement: Supplementary file 1 [file nutrients-14-02900-s001.zip › nutrients-1814922-supplementary.pdf]

Table S1. Omega-6 to omega-3 ratios according to diet group and sex

	Vegans	Omnivores	p-value
**Dietary omega-6 to omega-3 ratio**			
Total study population (n=72)	5.9:1 (4.5:1-7.5:1)	5.3:1 (4.5:1-7.3:1)	0.8
Women (n=36)	5.5:1 (4.6:1-8.5)[a]	6.4:1 (5.0:1-7.8:1)[b]	0.5
Men (n=36)	6.0:1 (4.3:1-7.2)[a]	4.8:1 (4.0:1-5.8:1)[b]	0.3
**Plasma omega-6 to omega-3 ratio**			
Total study population (n=72)	12.1:1 (10.5:1-14.6:1)	6.9:1 (6.0:1-8.3:1)	p<0.0001
Women (n=36)	12.7:1 (10.9:1-15.6:1)[a]	7.2:1 (6.0:1-8.2:1)[a]	p<0.0001
Men (n=36)	11.9:1 (9.8:1-14.4:1)[a]	6.6:1 (5.8:1-8.6:1)[a]	p<0.0001

[a] No statistical differences between men and women within diet group (p<0.5); [b] statistical differences between men and women within diet group (p=0.01)
